# Lumenal exposed regions of the D1 protein of PSII are long enough to be degraded by the chloroplast Deg1 protease

**DOI:** 10.1038/s41598-018-23578-x

**Published:** 2018-03-27

**Authors:** Ronit Rimon Knopf, Zach Adam

**Affiliations:** 1 The Robert H. Smith Institute of Plant Sciences and Genetics in Agriculture, Faculty of Agriculture, Food and Environment, The Hebrew University of Jerusalem, Rehovot, 76100 Israel; 2 Present Address: Evogene Ltd., Rehovot, 76120 Israel

## Abstract

Degradation of the D1 protein of photosystem II (PSII) reaction center is a pre-requisite for the repair cycle from photoinhibition. Two types of thylakoid proteases, FtsH and Deg, have been demonstrated to participate in this process. However, the location of the proteolytic sites of the lumenal Deg1 protease within its internal sphere raised the question whether the lumenal-exposed regions of D1 are indeed long enough to reach these sites. Implanting these regions into the stable GFP rendered it sensitive to the presence of Deg1 *in vitro*, demonstrating that the flexible regions of D1 that protrude into the lumen can penetrate through the three side-openings of Deg1 and reach its internal proteolytic sites. This mode of action, facilitating cooperation between proteases on both sides of the thylakoid membranes, should be applicable to the degradation of other integral thylakoid membrane proteins as well.

## Introduction

The thylakoid membrane houses the heart of the photosynthetic machinery, composed of four major complexes: photosystem I and photosystem II (PSII), capable of harvesting light energy and converting it into a chemical one, the cytochrome *b*_6_*-f* complex linking the two photosystems, and the ATP synthase. Their concerted action provides the chloroplast with NADPH and ATP, the reducing power and energy source, respectively, necessary for CO_2_ fixation. Most of the subunits of these multi-protein complexes are integral membrane proteins. Proper operation of this machinery under changing environmental conditions requires turnover of different subunits of these complexes at different rates. Thus, proteolytic degradation of specific chloroplast proteins is inherent to the capability of plants to maintain photosynthesis under different physiological conditions.

The most studied proteolytic event in the thylakoid membrane is that of the D1 protein of PSII, in the context of PSII repair from oxidative damage incurred by exposure to light that may lead to a phenomenon known as photoinhibition (for reviews, see^1,2^). This degradation of the D1 protein is a prerequisite for the incorporation of a newly synthesized D1 into the damaged complex and reactivation of PSII. As other thylakoid membrane proteins are also prone to irreversible oxidation by reactive oxygen species, although at a lower rate compared to D1, they are also subjected to proteolytic degradation. One of the preventive mechanisms to minimize the risk of oxidation damage induced by high light intensities, and hence of photoinhibition, involves adjustment of the size of the photosynthetic antenna, by degradation of chlorophyll binding proteins, which leads to a decrease in the excitation pressure. Another example of selective proteolytic degradation of a photosynthetic complex is that of the cytochrome *b*_6_*-f* complex, during the response of the chloroplast to nutrient deficiency^3^.

In the cases of the D1 protein and the cytochrome *b*_6_*-f* complex, a crucial role in the degradation has been assigned to the thylakoid membrane-bound, ATP-dependent FtsH metalloprotease complex, whose proteolytic domain is facing the stroma^4^. It is believed that the ATPase domain of FtsH is responsible for substrate recognition, and by virtue of its chaperone activity, the substrate is unfolded and fed into the proteolytic chamber. As degradation of integral membrane proteins involves extraction of trans-membrane helices from the hydrophobic core of the membrane, this could be facilitated by initial cleavage of the substrate to shorter fragments. At least in the case of D1 protein, proteases of the Deg family, associated with both the stroma and lumen sides of the thylakoid membrane, have been suggested to play a supportive role by cleaving hydrophilic segments of the D1 protein^5,6^.

**Figure 1:**
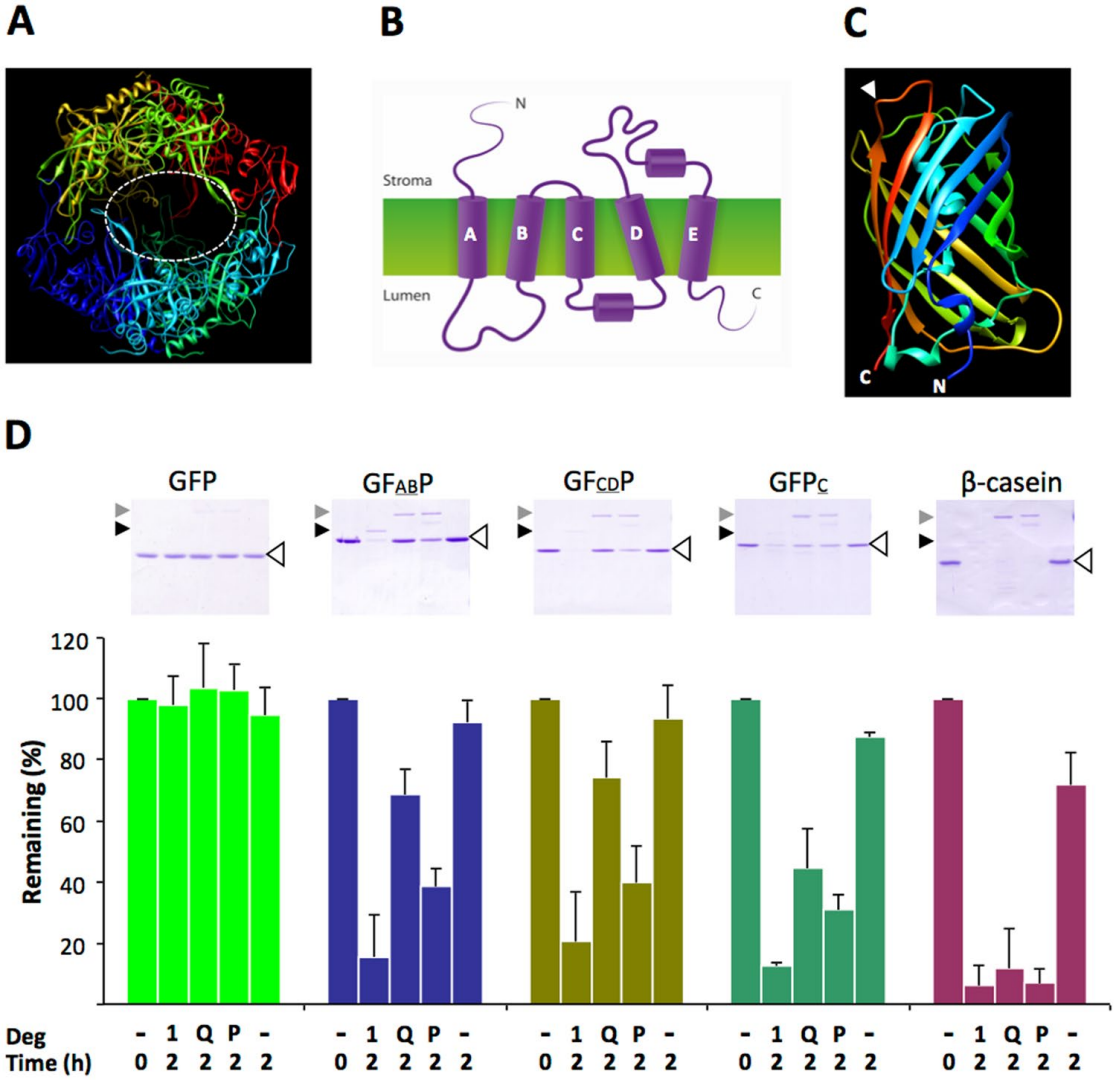
Analysis of Degradation of GFP-D1 Chimeric Proteins by Deg Proteases. (**A**) Crystal structure of Arabidopsis Deg1 hexamer at 2.5-Å resolution, adopted from PDB accession code 3QO6^9^. Each monomer is depicted in a different color. The upper and lower trimers, facing each other, are presented. One of the three side openings is surrounded by a white dashed line. The proteolytic sites are facing the inside of this cage-like structure. (**B**) Schematic presentation of the topology of the D1 protein of PSII in the thylakoid membrane. The amino- (N) and carboxy- (**C**) termini are exposed to the stroma and the thylakoid lumen, respectively. The hydrophobic core of the thylakoid membrane is colored in green and the five trans-membranes helices (**A-E**) are denoted. (**C**) Structure of GFP (PDB accession code 1GFL). The point of insertion of the hydrophilic loops AB and CD, between Pro211 and Asn212, is indicated by a white arrowhead at the top of the picture. The C-terminus of GFP, where the C-terminal segment of D1 was fused, as well as the N-terminus, are indicated at the bottom. (**D**) *In vitro* proteolytic degradation assay by Deg proteases. 20 μl reaction mixtures included 50 pmol of substrates (indicated by open arrowheads) and 5 pmol proteases (grey arrowheads point to the location of DegP and DegQ on the gel, and the black arrowhead indicates the location of Deg1), in 50 mM MES, pH 6.0, and incubated at 37 °C for 2 hrs. Reactions were terminated by adding SDS-solubilization buffer and resolved by SDS-PAGE (top panels). Substrate bands were quantified by scanning densitometry. Averages of the amount of substrates remaining in the mixture after 2 h incubation, from three experiments (±SD), are presented at the lower panel. GFABP and GFCDP are fusion proteins containing the respective loops in the middle of the GFP molecule (see panel C) and GFPC is a C-terminal extension of GFP. 1, Q and P below the bar graph refer to Deg1, DegQ and DegP, respectively.

Deg proteases are ATP-independent serine-type proteases of prokaryotic origin^7^. *E. coli* has three such proteases, DegP, DegQ and DegS, all located in the periplasmic space. Of the 16 Deg genes in the Arabidopsis genome, the products of three, Deg1, Deg5 and Deg8, are located in the thylakoid lumen, Deg2 and Deg7 are in the stroma, and others are found in mitochondria, peroxisome and the nucleus^8^. Whereas Deg5 and Deg8 have been suggested to interact with each other^6^, the active form of Deg1 is a homo-hexamer. The crystal structure of this protease, resolved at 2.5 Å, revealed a dimer of trimers, whose active sites (comprised of His173, Asp203 and Ser282) are located within a cage-like structure^9^ (Fig. 1A). Oligomerization of Deg1 monomers is pH-dependent and requires the protonation of His244. As a result, Deg1 is active at acidic pH, whereas in higher pH it is found in its monomeric inactive form^9^. Three pores, each 22 × 33 Å^2^ in size (see Fig. 1A), preclude the possibility of degrading tightly folded globular proteins, and restrict access to the active sites to unfolded proteins and extended polypeptide loop structures.

The dimensions of these pores and their distance from the proteolytic active sites (30–40 Å, see Supp. Fig. 4 in^9^) raised the question whether hydrophilic segments of the D1 protein, extruding into the lumen (see schematic presentation in Fig. 1B), are long enough to be degraded within the cage-like hexamer of Deg1. To answer this question, we generated fusion proteins, incorporating hydrophilic segments of the D1 protein, which are normally facing the lumen side of the thylakoid membrane, into GFP, and used them as substrates in *in vitro* proteolytic assays with different Deg proteases. We reasoned that the rigid structure of GFP (Fig. 1C) would mimic that of the hydrophobic region of the PSII complex, allowing flexibility of the hydrophilic segments of D1, similar to their *in vivo* situation in the lumen under conditions of PSII repair (which requires partial disassembly of PSII).

The lumen exposed, hydrophilic regions of D1 are the AB loop (Pro56 – Gly110) connecting the first and second trans-membrane helices, the CD loop (Gln165 – Leu193) connecting the third and fourth helices, and the C-terminus of D1 (Asn296 – Ala344) (see Fig. 1B). Both loops were inserted between Pro211 and Asn212 in the short loop (Lys209 – Asp216) connecting β-sheets 10 and 11 of GFP, and the D1 C-terminus was fused to that of GFP (Fig. 1C). The corresponding DNA constructs were expressed in *E. coli*, and the resulting His-tagged proteins were affinity-purified on Ni-NTA columns. These three proteins, together with His-tagged GFP and β-casein as controls, were used as substrates in the proteolytic assay. The enzymes used in the assay were recombinant *Arabidopsis thaliana* Deg1^10^, and DegP and DegQ of *E. coli*^7^.

The reaction mixture of the proteolytic assay contained 50 pmol of the substrates and 10-fold less of the different proteases. After incubation, the reaction mixtures were resolved by SDS-PAGE and the remaining substrate bands were quantified by scanning densitometry. Representative gels are shown at the top of Fig. 1D and averages of quantification of three experiments are at the bottom. The plant Deg1 and the two bacterial enzymes were equally active on the model substrate β-casein. In contrast, the tightly folded structure of GFP rendered it insensitivity to the presence of these proteases. Incorporating either the AB or CD loops, or the C-terminus of the D1 protein into GFP made the chimeric proteins sensitive to proteolysis (Fig. 1D). It appears that of the three enzymes, the plant Deg1 protease was the most efficient enzyme in degrading the fusion substrates, followed by DegP and DegQ of *E. coli*. These results demonstrate that the unfolded loops of D1 protruding out of the tightly folded cylinder-like structure of the GFP, as well as the extension at its C-terminus, are long enough to penetrate through the side pores of Deg1 and reach its active sites, which, as mentioned above, are 30–40 Å away. Since we could not detect any distinct fragments of the substrates even at shorter incubation times, we suggest that once an initial cleavage has occurred, the substrate GFP unfolds and is immediately degraded into very short peptides or free amino acids, consistent with the processive nature of Deg proteases^7^. These results support the notion that the segments of the D1 protein that are exposed to the lumen can be targeted and cleaved by the Deg1 protease.

In the fully assembled PSII complex, the segments of the D1 protein that are oriented towards the lumen are normally shielded by the oxygen-evolving complex (OEC) that is peripherally attached to the PSII core complex. In this conformation, these segments are protected from proteolysis by Deg1 or any other lumenal protease. This implies that a prerequisite for their cleavage by Deg1 is the detachment of OEC from the holo-PSII complex. Thus, proteolytic attack on the lumenal side of D1 is regulated at two levels: at the substrate level – partial disassembly of PSII, likely induced by oxidative damage, that exposes hydrophilic segments of D1 to the lumen; at the protease level – activation of Deg1 by its hexamerization, that is induced by acidification of the lumen. As both oxidative damage and lumen acidification result from the exposure of the photosynthetic machinery to light, the two levels of regulation are synchronized.

Although the D1 protein of PSII is one of the fastest turning over proteins in the chloroplast^11^, it is not the only thylakoid membrane protein that undergoes proteolytic degradation. All integral membrane proteins are anchored to the membrane by at least one trans-membrane helix, and most of them contain hydrophilic segments protruding to the lumen. We propose that, similar to the D1 protein, these segments might be targets of Deg1 protease, facilitating complete degradation of thylakoid membrane proteins, in cooperation with proteases on the stromal side of the thylakoid membrane, such as the FtsH complex.

## Methods

DNA sequences encoding the lumenal-exposed regions of the D1 protein from *Arabidopsis thaliana* (AtCg00020) (see Fig. 1B) were fused with the sequence encoding GFP **(**P42212**)**. The AB loop (Pro56 – Gly110) and the CD loop (Gln165 – Leu193) were inserted between Pro211 and Asn212 of GFP, and the C-terminus of D1 (Asn296 – Ala344) was fused downstream of that of GFP. All constructs contained 6xHis tag at their C-terminus. These constructs, as well as those encoding His-tagged Deg1 from *Arabidopsis thaliana*^10^ and DegP and DegQ from *E. coli*^7^, were expressed in bacterial cells, and affinity purified on Ni-NTA columns, as previously described^10^. Proteolytic degradation assays were conducted essentially as in^10^. Briefly, 50 pmol of substrates and 5 pmol of proteases were mixed in 50 mM MES, pH 6.0, in a total volume of 20 μl, and incubated at 37 °C for 2 hrs. The reactions were terminated by adding SDS-solubilization buffer and resolved by SDS-PAGE, and the remaining substrate bands were quantified by scanning densitometry.
